# The Services of Chemistry to Pharmacy

**Published:** 1876-03

**Authors:** 


### *THE SERVICES OF CHEMISTRY TO PHARMACY.*

With the eighteenth century is connected the birth of modern chemistry; and, while Priestly and Lavoisier are honored as having given a new impulsion to chemical theory, the Swedish apothecary Scheele will always be remembered as one who probably enriched the science with more discoveries than either of them. The three brightest names on the roll of great chemists in our century have been gathered from the ranks of the parmaceutical profession, viz., Davy, Liebig, and Dumas. But the debt owed by chemistry to pharmacy has been amply repaid: the labors of the chemist have transformed the pharmaceutical art, replacing empiricism by science, enriching the materia medica with a vast number of new substances, and introducing new processes. Such old-fashioned drugs as coral, egg-shells, and the like, were shown by the chemist to possess no other value than belongs to the calcareous salts of which they are chiefly composed. Iodine was shown to be the active principle in the drug, calcined sponge; and henceforth iodine takes the place of the crude and bulky residue from the burning of sponge. In like manner quinine and morphine replaced cinchona bark and opium.—*From Miscellany, in Popular Science Monthly for February.*

---

"Nelaton's Suppository" is a positive cure for Piles and Constipation, and Van Deren's Remedy will cure all cases of *Cholera Infantum and Bowel Complaints of Children.* Both are sold by all Druggists. Price 50 cents a package. Sent free by mail on receipt of price. Hall & Ruckel, Wholesale Druggists, 218 & 220 Greenwich St., New York.

